# Concurrent Detection of Varicella-Zoster Virus and Human Herpesvirus-6 in Corneal Endotheliitis: A Case Report

**DOI:** 10.7759/cureus.92242

**Published:** 2025-09-13

**Authors:** Minori Minamide, Hideki Fukuoka, Yu Fujimoto, Chie Sotozono

**Affiliations:** 1 Department of Ophthalmology, Kyoto Prefectural University of Medicine, Kyoto, JPN

**Keywords:** antiviral therapy, aqueous humor pcr, corneal endotheliitis, dual viral infection, endothelial cell loss, human herpesvirus-6, varicella-zoster virus

## Abstract

Viral corneal endotheliitis is predominantly caused by herpes viruses, with cytomegalovirus (CMV), herpes simplex virus (HSV), and varicella-zoster virus (VZV) being the most commonly implicated pathogens. The simultaneous detection of multiple herpes viruses in aqueous humor by polymerase chain reaction (PCR) is a rare phenomenon that presents diagnostic and therapeutic challenges. We report a unique case of a 75-year-old male patient who developed unilateral corneal endotheliitis with concurrent detection of VZV and human herpesvirus-6 (HHV-6) in aqueous humor PCR. The patient had a history of herpes zoster ophthalmicus with conjunctivitis six months prior to presentation. Initial treatment with acyclovir ointment and betamethasone drops was supplemented with ganciclovir drops targeting HHV-6. The patient demonstrated complete clinical recovery with viral clearance at two months, though endothelial cell count remained reduced at 917 cells/mm² at three months, reflecting permanent endothelial damage. No recurrence was observed during one year of follow-up. This, to the best of our knowledge, represents the first reported case of concurrent VZV and HHV-6 detection in viral corneal endotheliitis. The successful therapeutic response to combined antiviral therapy targeting both viral pathogens suggests that comprehensive herpes virus screening and tailored antiviral treatment may be crucial for optimal outcomes. Digital PCR technology enables precise viral identification, facilitating evidence-based antiviral selection in complex cases.

## Introduction

Viral corneal endotheliitis represents a significant cause of corneal endothelial dysfunction and vision-threatening complications. The condition is predominantly caused by members of the herpes virus family, which demonstrate the ability to establish latent infections with periodic reactivation. The most frequently implicated pathogens include cytomegalovirus (CMV) [1], herpes simplex virus (HSV) [2], and varicella-zoster virus (VZV) [3]. Less commonly, other viruses such as Epstein-Barr virus (EBV) [4], human herpesvirus-6 (HHV-6) [5], human herpesvirus-7 (HHV-7) [6], human herpesvirus-8 (HHV-8) [7], and mumps virus [8] have been reported as causative agents.

The simultaneous detection of multiple herpes viruses in aqueous humor from patients with corneal endotheliitis is an uncommon phenomenon that has been sporadically reported in the literature. Previous case reports have documented concurrent detection of CMV and HHV-6 [9], as well as HSV-1 and HHV-6 [10], in unilateral corneal endotheliitis. The pathogenetic mechanisms underlying dual viral infections remain poorly understood, with theories including primary viral infection followed by secondary viral reactivation or simultaneous reactivation of multiple latent viruses.

HHV-6, a β-herpesvirus that establishes latency in monocytes and bone marrow progenitor cells, has been increasingly recognized as a pathogen in ocular inflammatory diseases. Studies investigating HHV-6 detection in ocular inflammatory conditions have revealed its presence in various forms of uveitis, endophthalmitis, and keratitis [11]. The virus has been shown to possess pathogenic potential for corneal endothelium, with reports of HHV-6-induced corneal endotheliitis responding to ganciclovir therapy [5].

To our knowledge, no cases of concurrent VZV and HHV-6 detection in corneal endotheliitis have been reported in the peer-reviewed literature. We present a case of unilateral corneal endotheliitis with simultaneous detection of VZV and HHV-6 in aqueous humor PCR that responded favorably to combined antiviral therapy targeting both viral pathogens.

## Case presentation

A 75-year-old male presented with blurred vision in his right eye. His medical history was significant for cerebrovascular accident and, notably, herpes zoster ophthalmicus with associated conjunctivitis affecting the right eye six months prior to the current presentation. The patient had no history of immunocompromise, corticosteroid use, or previous intraocular surgery.

The patient was initially evaluated at another ophthalmology facility, where a comprehensive examination revealed corneal endothelial dysfunction and significant corneal edema in the right eye. Based on the clinical presentation and history of herpes zoster ophthalmicus, presumptive viral corneal endotheliitis was diagnosed. Initial treatment was instituted with acyclovir ophthalmic ointment (five times daily), 0.1% betamethasone phosphate eye drops (four times daily), and oral famciclovir (1000 mg daily).

Given the severity of the corneal endotheliitis and the need for a definitive viral diagnosis, aqueous humor paracentesis was performed for polymerase chain reaction (PCR) analysis. The PCR testing revealed positive results for both VZV and HHV-6 DNA, prompting referral to our tertiary care institution for specialized management of this unusual dual viral infection.

**Figure 1:**
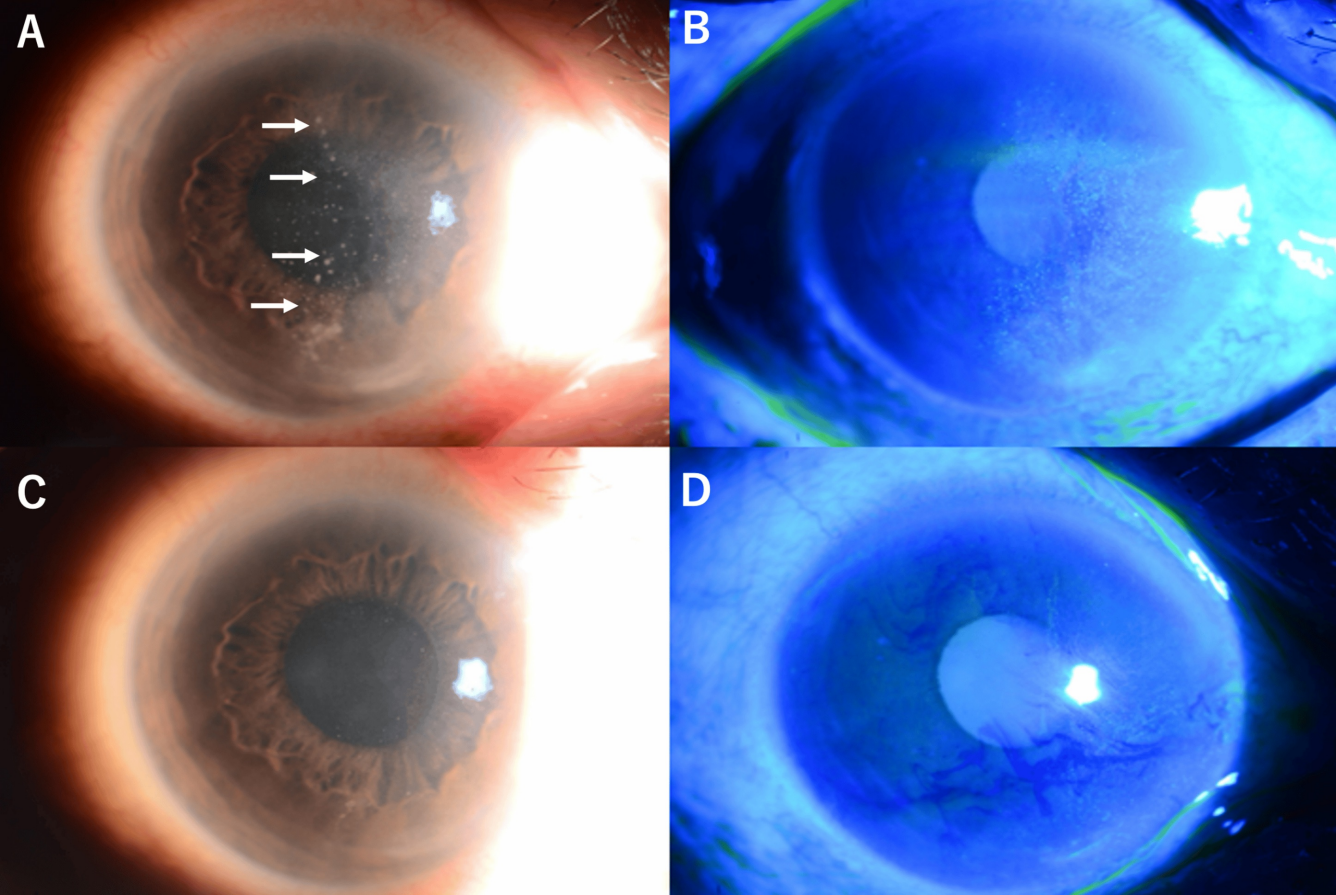
Anterior segment photographs demonstrating clinical progression of VZV and HHV-6 corneal endotheliitis. (A) Initial presentation showed multiple keratic precipitates (white arrows) distributed across the corneal endothelium with significant corneal edema; (B) Fluorescein staining at initial presentation demonstrated significant corneal edema; (C) Ten-day follow-up examination demonstrated marked reduction in keratic precipitates and improvement in corneal transparency following initiation of combined antiviral therapy; (D) Fluorescein staining at 10-day follow-up showed improvement in corneal edema. VZV: varicella-zoster virus; HHV-6: human herpesvirus-6

Upon presentation to our institution, his best-corrected visual acuity was 0.4 (20/50) in the right eye and 1.5 (20/13) in the left eye. Intraocular pressure measured 18 mmHg in both eyes. Slit-lamp examination of the right eye revealed characteristic findings of viral corneal endotheliitis, including multiple keratic precipitates distributed across the corneal endothelium and significant corneal edema affecting visual clarity. The anterior chamber showed a mild inflammatory reaction without hypopyon formation (Figures 1A, 1B).

The left eye demonstrated normal anterior segment architecture with no evidence of inflammation or endothelial abnormalities. Fundoscopic examination revealed normal posterior segment findings in both eyes, with no evidence of retinal involvement or optic nerve abnormalities.

**Figure 2:**
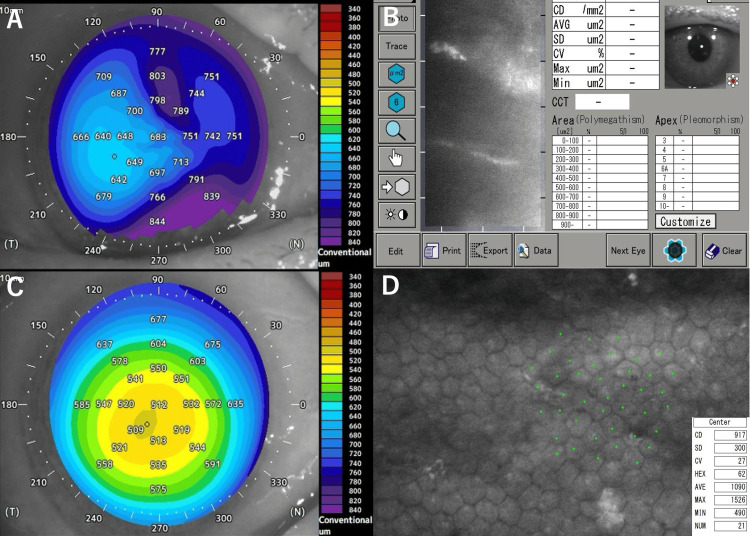
Anterior segment optical coherence tomography (OCT) and specular microscopy findings demonstrating corneal structural changes. (A) Initial anterior segment OCT revealed markedly increased central corneal thickness of 683 μm due to severe corneal edema; (B) Initial non-contact specular microscopy showed an inability to visualize endothelial cells due to severe corneal edema; (C) Three-month follow-up anterior segment OCT demonstrated normalized central corneal thickness of 512 μm following successful antiviral therapy; (D) Three-month follow-up contact specular microscopy revealed endothelial cell density of 917 cells/mm². Enlarged endothelial cells, larger than normal, were observed, consistent with compensatory changes following cell loss.

Central corneal thickness was markedly increased at 683 μm as measured by anterior segment optical coherence tomography (Casia 2; Tomey Corporation, Nagoya, Japan) (Figure 2A).

Endothelial cell density (ECD) could not be measured with non-contact specular microscopy due to severe corneal edema (Figure 2B).

Based on the clinical presentation, diagnostic imaging, and microbiologic confirmation, a diagnosis of concurrent VZV and HHV-6 corneal endotheliitis was established. Given the dual viral etiology, a comprehensive antiviral treatment regimen was implemented targeting both pathogens. The primary antiviral therapy consisted of acyclovir ophthalmic ointment 3% administered five times daily targeting VZV and ganciclovir ophthalmic 0.5% eye drops administered eight times daily targeting HHV-6. Anti-inflammatory therapy included betamethasone phosphate 0.1% eye drops, which were reduced from four times daily to twice daily to minimize the potential for enhancing viral replication. Prophylactic antibiotic therapy with levofloxacin 1.5% eye drops was administered two times daily as a preventive measure against secondary bacterial infection.

At the 10-day follow-up, the patient demonstrated encouraging clinical improvement, with visual acuity improving to 0.5 (20/40) in the right eye. Intraocular pressure remained stable at 14 mmHg in the right eye and 15 mmHg in the left eye. Slit-lamp examination revealed a reduction in corneal edema and keratic precipitates, indicating a positive treatment response (Figure 1C, 1D).

At the two-month follow-up, significant clinical improvement was observed, with visual acuity reaching 1.0 (20/20) in the right eye. Intraocular pressure was 13 mmHg in the right eye and 15 mmHg in the left eye. Central corneal thickness was normalized to 512 μm. Most importantly, repeated aqueous humor PCR analysis demonstrated complete clearance of both VZV and HHV-6 DNA, confirming successful antiviral therapy.

At the three-month follow-up, after gradual tapering of topical medications, the patient maintained excellent visual acuity of 1.2 (20/16) in the right eye. Intraocular pressure remained stable at 13 mmHg in the right eye and 14 mmHg in the left eye. Central corneal thickness was 512 μm (Figure 2C). Specular microscopy, now feasible due to corneal clearing, revealed an ECD of 917 cells/mm² in the right eye (Figure 2D), reflecting permanent endothelial cell loss secondary to the viral infection.

At the 16-month follow-up, the ECD in the right eye had increased to 2020 cells/mm², suggesting migration of normal endothelial cells to areas where endothelial cells had been lost due to the infection.

During long-term follow-up, the patient remained recurrence-free throughout one year of follow-up, with stable visual acuity and no evidence of ongoing inflammation or viral reactivation.

## Discussion

Herpes virus classification and ocular tropism

HHVs comprise eight distinct viruses classified into three subfamilies based on their biological properties, cellular tropism, and sites of latency establishment [12]. The α-herpesviruses represent neurotropic pathogens that demonstrate a preferential affinity for neural tissue. Within this subfamily, HSV-1 establishes latency in the trigeminal ganglia, HSV-2 maintains dormancy in the sacral nerve plexus, and VZV establishes latent infection in both the trigeminal and dorsal root ganglia.

The β-herpesviruses constitute lymphotropic viruses with distinct cellular preferences for hematopoietic lineages. CMV establishes latency in bone marrow progenitor cells and monocytes, while HHV-6 similarly maintains dormancy in monocytes and bone marrow progenitor cells. In contrast, HHV-7 exhibits a specific tropism for CD4+ T cells, thereby establishing its latent reservoir within this specialized lymphocyte population.

The γ-herpesviruses are lymphotropic pathogens that primarily target B lymphocytes for establishing latency. Epstein-Barr virus establishes persistent infection in B cells, while HHV-8, also known as Kaposi sarcoma-associated herpesvirus, similarly maintains latency in B cells. This classification system provides a framework for understanding the diverse pathogenetic mechanisms and clinical manifestations associated with herpes virus infections, particularly in the context of ocular inflammatory diseases.

Hypothesized pathogenetic mechanisms of dual viral infection

The concurrent detection of VZV and HHV-6, which have distinct cellular tropism and latency sites, raises intriguing questions regarding the underlying pathogenetic mechanisms. Several hypotheses may explain this phenomenon.

The sequential viral reactivation hypothesis suggests that VZV, with its known predilection for trigeminal ganglia and propensity for reactivation along the ophthalmic division, may have served as the primary pathogen initiating corneal endothelial inflammation. The resulting inflammatory milieu may have subsequently facilitated secondary reactivation of HHV-6 from its latent reservoirs in circulating monocytes and bone marrow progenitor cells. This cascade effect would explain the temporal relationship between the initial inflammatory insult and the detection of multiple viral pathogens within the anterior chamber.

Alternatively, the simultaneous reactivation hypothesis proposes that both viruses may have undergone concurrent reactivation triggered by common predisposing factors such as advanced age, immunosenescence, or systemic physiological stress. In this scenario, both pathogens would contribute independently to the pathogenesis of corneal endotheliitis, with each virus targeting corneal endothelial cells through distinct but potentially synergistic mechanisms. This dual viral assault on the corneal endothelium could result in more severe tissue damage and a prolonged inflammatory response compared to single-pathogen infections.

HHV-6 in ocular inflammatory diseases

The pathogenic role of HHV-6 in ocular inflammatory diseases has been increasingly recognized through molecular diagnostic advances. Sugita et al. conducted a comprehensive study investigating HHV-6 detection rates in ocular inflammatory diseases, revealing HHV-6 presence in seven of 350 patients (2%) with uveitis or endophthalmitis and one of 65 patients (1.5%) with keratitis [11]. Importantly, their detection of HHV-6 mRNA in vitreous samples from patients with ocular toxocariasis provided evidence that HHV-6 can actively replicate within the ocular environment.

The study demonstrated that HHV-6, which establishes latency in lymphoid and myeloid cell populations, may enter inflamed ocular tissues through recruitment of infected immune cells and subsequently undergo local reactivation and replication. This mechanism supports the hypothesis that HHV-6 detection in our case may represent secondary involvement following primary VZV-induced inflammation.

However, the documented cases of HHV-6 as a sole pathogen in corneal endotheliitis [5] indicate that this virus possesses independent pathogenic potential for corneal endothelium, suggesting that it may contribute directly to disease pathogenesis rather than serving merely as an opportunistic bystander.

Therapeutic considerations and antiviral selection

The selection of appropriate antiviral therapy for herpes virus-induced corneal endotheliitis requires a comprehensive understanding of viral subfamily characteristics and corresponding drug susceptibility patterns (Table 1).

**Table 1:** Clinical effectiveness of acyclovir and ganciclovir against herpes viruses +++: highly effective, first-line therapy with demonstrated superior clinical response; ++: moderately effective, alternative or adjunctive therapy with good clinical response; +: mildly effective, limited efficacy with modest clinical benefit; -: ineffective, minimal or no demonstrated clinical benefit HSV: herpes simplex virus; VZV: varicella-zoster virus; CMV: cytomegalovirus; HHV: human herpesvirus; EBV: Epstein-Barr virus Source: [13-17]

Subfamily	Virus type	Acyclovir	Ganciclovir
a	HSV-1	+++	++
	HSV-2	+++	++
	VZV(HHV-3)	+++	++
b	CMV(HHV-5)	-	+++
	HHV-6	-	+++
	HHV-7	-	-
g	EBV(HHV-4)	++	++
	HHV-8	-	++

The α-herpesvirus subfamily, comprising HSV-1, HSV-2, and VZV, demonstrates excellent response to acyclovir, which serves as the first-line therapeutic agent. These viruses share similar thymidine kinase enzyme systems that effectively activate acyclovir, resulting in selective viral DNA synthesis inhibition [13].

In contrast, β-herpesviruses, including CMV and HHV-6, exhibit superior susceptibility to ganciclovir compared to acyclovir [14]. The fundamental difference in viral enzyme systems between α- and β-herpesviruses necessitates distinct therapeutic approaches. Notably, HHV-7 demonstrates resistance to both acyclovir and ganciclovir [15], presenting therapeutic challenges in confirmed cases of HHV-7-associated ocular inflammation.

The γ-herpesvirus subfamily presents variable therapeutic responses. EBV demonstrates moderate susceptibility to both acyclovir and ganciclovir [16], while HHV-8 shows a selective response to ganciclovir therapy [17].

Clinical significance of endothelial cell loss

The documented endothelial cell count of 917 cells/mm² at three months represents a critical finding that underscores the potentially devastating consequences of viral corneal endotheliitis. This degree of endothelial cell loss places the patient at significant risk for future corneal decompensation, particularly given that corneal endothelial cells have limited regenerative capacity. The finding emphasizes the importance of early diagnosis and aggressive antiviral therapy to minimize irreversible endothelial damage.

## Conclusions

We report, to the best of our knowledge, the first documented case of corneal endotheliitis with concurrent VZV and HHV-6 detection in aqueous humor PCR analysis. The successful viral clearance with combined acyclovir and ganciclovir therapy suggests both viruses contributed actively to disease pathogenesis. However, significant endothelial cell loss at three months emphasizes the potentially irreversible consequences and critical importance of early aggressive treatment in viral corneal endotheliitis.

This case highlights the evolving role of molecular diagnostics in ophthalmology and the need for comprehensive herpes virus screening in corneal endotheliitis. Future research should focus on establishing optimal treatment protocols for dual viral infections and developing strategies to minimize irreversible endothelial damage in viral corneal endotheliitis.
